# High-resolution crystal structures of the solubilized domain of porcine cytochrome *b*_5_

**DOI:** 10.1107/S1399004715009438

**Published:** 2015-06-30

**Authors:** Yu Hirano, Shigenobu Kimura, Taro Tamada

**Affiliations:** a Quantum Beam Science Center, Japan Atomic Energy Agency, 2-4 Shirakata, Tokai, Ibaraki 319-1195, Japan; b Department of Biomolecular Functional Engineering, Faculty of Engineering, Ibaraki University, 4-12-1 Nakanarusawa, Hitachi, Ibaraki 316-8511, Japan

**Keywords:** electron transfer, haem, sub-angstrom resolution

## Abstract

Mammalian microsomal cytochrome *b*_5_ has multiple electron-transfer partners that function in various electron-transfer reactions. Four crystal structures of the solubilized haem-binding domain of cytochrome *b*_5_ from porcine liver were determined at sub-angstrom resolution (0.76–0.95 Å) in two crystal forms for both the oxidized and reduced states. The high-resolution structures clearly displayed the electron density of H atoms in some amino-acid residues. Unrestrained refinement of bond lengths revealed that the protonation states of the haem propionate group may be involved in regulation of the haem redox properties. The haem Fe coordination geometry did not show significant differences between the oxidized and reduced structures. However, structural differences between the oxidized and reduced states were observed in the hydrogen-bond network around the axial ligand His68. The hydrogen-bond network could be involved in regulating the redox states of the haem group.

## Abstract 2

Crystal structures of the solubilized domain of cytochrome *b*_5_ from porcine liver were determined at sub-angstrom resolution in two crystal forms for both the oxidized and reduced states. The high-resolution structures provided information about the factors that are important for regulating the electronic properties of the haem group of cytochrome *b*_5_.

## 1. Introduction   

Mammalian microsomal cytochrome *b*_5_ is anchored to the membrane of the endoplasmic reticulum and is involved in various electron-transfer reactions, such as lipid unsaturation, cholesterol synthesis and drug metabolism. Because of its diversity of function, cytochrome *b*_5_ has been reported to interact with multiple electron-transfer partners including NADH-cytochrome *b*_5_ reductase (b5R; Spatz & Strittmatter, 1973 ▸), C4-methyl sterol oxidase (Fukushima *et al.*, 1981 ▸), cytochrome *c* (Ren *et al.*, 2004 ▸), NADH-cytochrome P450 reductase and cytochrome P450 (Porter, 2002 ▸). Mammalian microsomal cytochrome *b*_5_ consists of 134 residues, in which the N-terminal ∼100 residues contain the haem-binding region located at the cytoplasmic side of the endoplasmic reticulum membrane and the C-terminal ∼30 residues contain the membrane-anchoring region and the signal sequence targeting the endoplasmic reticulum membrane (Mitoma & Ito, 1992 ▸). The haem Fe atom is coordinated by two axial ligands, His44 and His68, and the haem group is not bound covalently to the protein moiety. The numbering of amino-acid residues in this study is based on the cytochrome *b*_5_ coding region and is different from the numbering of the protease-solubilized cytochrome *b*_5_ from bovine liver reported previously (Durley & Mathews, 1996 ▸). For example, His44 and His68 in this study are His39 and His63 in the protease-solubilized bovine cytochrome *b*_5_.

The crystal structure of cytochrome *b*_5_ has been obtained at 1.5 Å resolution using the protease-solubilized haem-binding region from bovine liver (Durley & Mathews, 1996 ▸). The solution structures of the solubilized domain of cytochrome *b*_5_ (b5) have also been reported for several species including cattle (Zhang *et al.*, 2004 ▸), rat (Banci *et al.*, 1997 ▸; Arnesano *et al.*, 1998 ▸) and rabbit (Banci *et al.*, 2000 ▸). The solution structures of b5 have revealed heterogeneity in the orientation of the haem group. The haem group of rat b5 has two orientations related by a 180° rotation about the porphyrin α,γ-*meso* axis (Rivera *et al.*, 1992 ▸; Altuve *et al.*, 2004 ▸), although one of the two orientations is predominantly observed in the haem group of bovine b5. Val28 of rat b5, as opposed to Leu28 of bovine b5, is located at the bottom of the haem-binding crevasse. Differences in the haem orientations are considered to be caused by steric repulsion between the hydrophobic residues and the vinyl group of the haem. However, it is still unclear how the orientation affects the electron-transfer reaction (Walker *et al.*, 1988 ▸). Solution structures of rat b5 have been solved in both the oxidized and reduced forms (Banci *et al.*, 1997 ▸; Arnesano *et al.*, 1998 ▸). A comparison of the average structures of both redox forms suggests that mobility around the haem group is important to optimize interactions with the electron-transfer partners.

Structural information on bovine b5 has been utilized for extensive mutation studies related to protein stability, haem redox potential and interaction with electron-transfer partners. Protein stability is decreased by the mutation of valine residues (Val50 and Val66) that are located at the haem edge and restrict the solvent-accessibility of the haem group (Gan *et al.*, 2002 ▸; Wu *et al.*, 2000 ▸). Stability is also decreased by the mutation of Phe63 involved in a π-stacking interaction with the axial ligand His68 (Wang *et al.*, 2006 ▸). The redox potential is decreased by the mutation of Phe40 located near the haem plane (Yao *et al.*, 2002 ▸) and by the mutation of Ser69 interacting with the haem propionate group (Funk *et al.*, 1990 ▸). The mutation of Val50 and Val66 also influences the redox potential of the haem (Gan *et al.*, 2002 ▸; Wu *et al.*, 2000 ▸). Mutations of the surface acidic residues (Glu49, Glu53, Glu61 and Asp65) decrease the association constant between b5 and cytochrome *c* (Sun *et al.*, 1999 ▸; Qian *et al.*, 2001 ▸; Wu *et al.*, 2001 ▸). Electrostatic interactions between these acidic residues and basic residues on the surface of cytochrome *c* have been thought to participate in formation of the b5–cytochrome *c* complex.

We have recently reported crystal structures of porcine b5R in both fully oxidized and reduced forms (Yamada *et al.*, 2013 ▸). The oxidized structure determined at 0.78 Å resolution includes H atoms that elucidate a hydrogen-bonding network around FAD. The reduced structure determined at 1.68 Å resolution shows a slight shift in the relative arrangement of the FAD- and NADH-binding domains compared with the oxidized structure. Although structural studies have been performed for several mammalian b5s, structural information has not yet been obtained for porcine b5. In addition, X-ray crystal structures determined at sub-angstrom resolution have not been obtained for both the oxidized and reduced forms of b5. The high-resolution structures of both redox forms are important for understanding the structural features involved in regulation of the electronic properties of the haem group. In this work, we report the crystal structures of porcine b5 determined at sub-angstrom resolutions in both the oxidized and reduced forms. The crystal structures were obtained for two crystal forms of both redox forms. A structural comparison was performed between the two redox forms and also between the two crystal forms. The comparison using high-resolution structures revealed that structural differences around the haem group may be important for regulating the electronic properties of the haem.

## 2. Experimental procedures   

### 2.1. Overexpression and purification   

The N-terminal solubilized domain of porcine cytochrome *b*_5_, consisting of amino-acid residues 1–94, was overexpressed in *Escherichia coli* BL21 cells containing plasmid pCPb5, as described previously (Kimura *et al.*, 2003 ▸). The purification procedure for porcine b5 has also been reported previously (Kimura *et al.*, 2003 ▸), but anion-exchange chromatography was additionally performed. The b5 fraction after size-exclusion chromatography was purified using a Resource Q column (GE Healthcare). Final samples were obtained in the oxidized form as deduced from the UV–Vis absorption spectrum and were concentrated to 30 mg ml^−1^ as calculated using an extinction coefficient of ∊_413_ = 1.13 × 10^5^ *M* ^−1^ cm^−1^. The reduced form of b5 was obtained by adding sodium dithionite to the oxidized form of b5 to a final concentration of 5 m*M*. To monitor the autoxidation of b5, UV–Vis absorption spectra were measured 0, 5, 10, 25, 60, 120 and 240 min after adding sodium dithionite under aerobic conditions at room temperature. The spectral measurements were performed three times.

### 2.2. Crystallization   

Initial screening of crystallization conditions was performed using the oxidized form of b5 with Crystal Screen, Crystal Screen 2 (Hampton Research) and Precipitant Synergy (Molecular Dimensions). Crystals were obtained under several conditions containing polyethylene glycol (PEG) as a precipitant. After optimization of the crystallization conditions, two crystal forms were obtained by the hanging-drop vapour-diffusion method at 293 K. Form 1 crystals were obtained by mixing 1 µl protein solution with 1 µl reservoir solution consisting of 24%(*w*/*v*) PEG 1500, 4%(*v*/*v*) 2-propanol, 0.1 *M* calcium chloride, 0.1 *M* HEPES–NaOH pH 7.5. The final pH of the reservoir solution was 7.43 at 298 K. Form 2 crystals were obtained by mixing 1 µl protein solution with 1 µl reservoir solution consisting of 15%(*v*/*v*) PEG 400, 3%(*w*/*v*) PEG 3350, 0.1 *M* sodium acetate pH 5.5. The final pH of the reservoir solution was 5.52 at 298 K. Crystallization of the reduced form of b5 was performed in an anaerobic chamber (Anaero Box ANX-3, Hirasawa). The reduced form of b5 was prepared by adding sodium dithionite to the oxidized form in the anaerobic chamber to a final concentration of 5 m*M*. Crystals of the reduced form of b5 were obtained under the same conditions as the oxidized form of b5.

### 2.3. Data collection   

Before diffraction data collection, crystals were transferred to cryoprotectant conditions. Form 1 crystals of the oxidized form were soaked in a cryoprotectant solution consisting of 35%(*w*/*v*) PEG 1500, 4%(*v*/*v*) 2-propanol, 0.1 *M* calcium chloride, 0.1 *M* HEPES–NaOH pH 7.5. Form 2 crystals of the oxidized form were soaked in a cryoprotectant solution consisting of 30%(*v*/*v*) PEG 400, 3%(*w*/*v*) PEG 3350, 0.1 *M* sodium acetate pH 5.5. Form 1 crystals of the reduced form were soaked in a cryoprotectant solution consisting of 35%(*w*/*v*) PEG 1500, 4%(*v*/*v*) 2-propanol, 0.1 *M* calcium chloride, 0.1 *M* HEPES–NaOH pH 7.5, 10 m*M* sodium dithionite in the anaerobic chamber. Form 2 crystals of the reduced form were soaked in a cryoprotectant solution consisting of 30%(*v*/*v*) PEG 400, 3%(*w*/*v*) PEG 3350, 0.1 *M* sodium acetate pH 5.5, 10 m*M* sodium dithionite in the anaerobic chamber. Crystals of the oxidized form were flash-cooled in a nitrogen-gas stream at 100 K just before data collection. Crystals of the reduced form were cooled in liquid nitrogen in the anaerobic chamber and stored until diffraction data collection.

Diffraction data sets were collected on the BL5A and BL17A beamlines at Photon Factory (PF), Tsukuba, Japan. Diffraction intensities were measured using ADSC Q315 (BL5A) and ADSC Q270 (BL17A) CCD detectors. Two data sets (high resolution and low resolution) were separately collected from a single crystal at different positions. For the high-resolution data sets, the positions at which the crystals were exposed to the X-rays were changed in order to restrict the maximum dose to 3.0 × 10^5^ Gy for each exposure position. Dose calculation was performed using *RADDOSE* (Paithankar *et al.*, 2009 ▸). For the low-resolution data sets, the crystals were exposed to attenuated X-rays to avoid overloading the CCD detectors. Diffraction data sets were integrated and scaled using the *HKL*-2000 program package (Otwinowski & Minor, 1997 ▸). The high- and low-resolution data sets were merged to obtain a complete data set.

### 2.4. Phasing and refinement   

The crystal structure of porcine b5 was determined using the molecular-replacement method with the crystal structure of bovine b5 (PDB entry 1cyo; Durley & Mathews, 1996 ▸) without the haem group or solvent molecules as a search model. One molecule was found in the asymmetric unit in both the form 1 and form 2 crystals. Structure refinement started with rigid-body refinement in the resolution range 8.0–1.2 Å using *PHENIX* (Adams *et al.*, 2010 ▸). Positional and isotropic displacement parameters were refined in the resolution range 50–1.2 Å. Manual model fitting and incorporation of the haem group were performed using *Coot* (Emsley *et al.*, 2010 ▸). Ordered water molecules were automatically included in the model when peak heights in the *F*_o_ − *F*_c_ map were detected at the 4.0σ level. Other solvent molecules were manually incorporated into the model. Anisotropic displacement parameters were then refined in the whole resolution range using *SHELXL* (Sheldrick, 2008 ▸). Restraints on bond angles and lengths were removed for the single-conformation residues and the haem group only when they converged to reasonable geometries after conjugate-gradient least-squares refinement. H atoms were incorporated in the model when they were observed in the hydrogen OMIT map contoured at the 1.5σ level. The bond lengths and angles of the H atoms were constrained using the riding model in *SHELXL*. The estimated standard deviations of bond angles and lengths were obtained by one cycle of full-matrix least-squares refinement. Figures were prepared using *PyMOL* (DeLano, 2002 ▸).

## 3. Results   

### 3.1. Structure of porcine b5   

An autoxidation experiment showed that 50% of the reduced form of b5 was converted to the oxidized form following a 30 min incubation (Supplementary Fig. S1). Therefore, the crystallization and cooling of crystals were performed in the anaerobic chamber for the reduced form. Oxidized and reduced forms of b5 were crystallized in two crystal forms, both of which belonged to the orthorhombic space group *P*2_1_2_1_2_1_, with unit-cell parameters *a* = 32.3, *b* = 39.3, *c* = 61.0 Å (oxidized form 1), *a* = 33.6, *b* = 51.2, *c* = 55.1 Å (oxidized form 2), *a* = 33.6, *b* = 38.9, *c* = 60.6 Å (reduced form 1) and *a* = 33.7, *b* = 51.3, *c* = 55.3 Å (reduced form 2). The crystal structures were determined at resolutions of 0.83 Å (oxidized form 1), 0.93 Å (oxidized form 2), 0.76 Å (reduced form 1) and 0.95 Å (reduced form 2). Diffraction data and refinement statistics are given in Table 1 ▸.

All structures included residues Ala8–Lys94 in the final models; the remaining seven residues at the N-terminus were not observed in the 2*F*_o_ − *F*_c_ electron-density maps. The final models contained 20–28 multiple conformations of amino-acid residues. Many multiple conformations were observed for the N-terminal (Ala8–Tyr11) and C-terminal (Lys91–Lys94) residues. 48% (oxidized form 1), 27% (oxidized form 2), 49% (reduced form 1) and 17% (reduced form 2) of the H atoms in the amino-acid residues could be included in the models. In the form 1 structures, electron density derived from H atoms was observed for some amino-acid residues. Electron density for H atoms bound to N atoms was observed as well as for those bound to C atoms. In the form 2 structure, H atoms were primarily added to atoms with low *B*_eq_ values. The final models of both crystal forms also contained some of the H atoms of the haem group (Table 1 ▸). Two Ca ions and one acetate ion were found in the structures of the form 1 and 2 crystals, respectively (Table 1 ▸).

**Figure 1:**
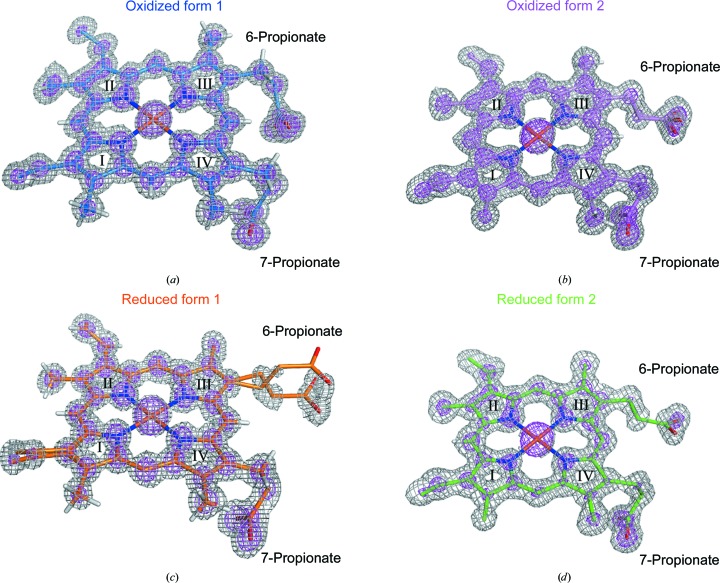
Electron density of the haem group. *F*_o_ − *F*_c_ haem OMIT maps are shown as grey and pink meshes contoured at the 3.0σ and 7.0σ levels, respectively, in the oxidized form 1 structure (*a*), in the oxidized form 2 structure (*b*), in the reduced form 1 structure (*c*) and in the reduced form 2 structure (*d*). The porphyrin-ring numbers and the positions of the 6-propionate and 7-propionate groups are indicated in the figures.

The orientation of the haem group was clearly confirmed by a haem OMIT map. Figs. 1 ▸(*a*)–1 ▸(*d*) show the OMIT maps for the haem molecules in the structures of oxidized form 1, oxidized form 2, reduced form 1 and reduced form 2, respectively. The haem orientation in porcine b5 corresponds to the major isomer observed in the rat b5 structure. The residue located at the bottom of the haem-binding crevasse is Leu28 in porcine b5, which is the same as that in bovine b5 (Supplementary Fig. S2).

**Figure 2:**
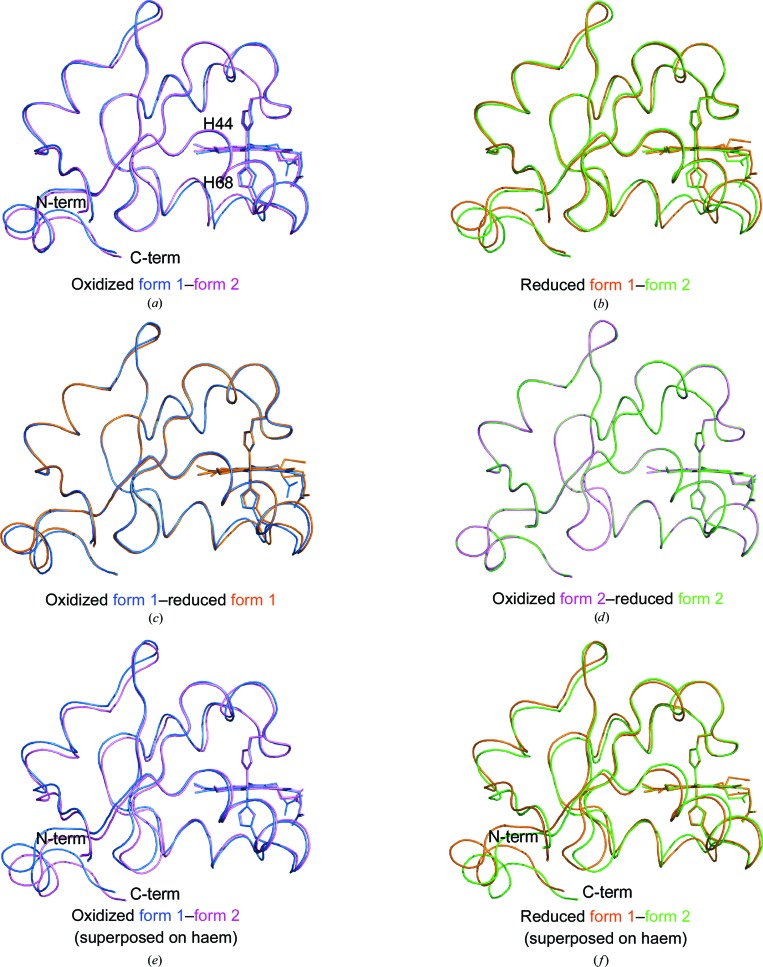
Structure comparison among the porcine b5 structures. (*a*) Superposition of C^α^ atoms between the form 1 (blue) and form 2 (pink) structures of the oxidized forms. (*b*) Superposition of C^α^ atoms between the form 1 (orange) and form 2 (green) structures of the reduced forms. (*c*) Superposition of C^α^ atoms between the oxidized (blue) and reduced (orange) forms of the form 1 structures. (*d*) Superposition of C^α^ atoms between the oxidized (pink) and reduced (green) forms of the form 2 structures. (*e*) Superposition of the atoms in the porphyrin rings between the form 1 (blue) and form 2 (pink) structures of the oxidized forms. (*f*) Superposition of the atoms in the porphyrin rings between the form 1 (orange) and form 2 (green) structures of the reduced forms.

All four structures exhibited high similarity to each other (Figs. 2 ▸ *a*–2 ▸ *d*). Superposition of all C^α^ atoms showed root-mean-square distances between the form 1 and form 2 structures of 0.51–0.67 Å, which were larger than those between the oxidized and reduced structures in the same crystal form (0.11 and 0.31 Å) (Supplementary Table S1). When the structures of the two crystal forms were superposed on their haem-plane atoms, both the oxidized and reduced structures showed a highly similar structure around the haem groups (Figs. 2 ▸ *e* and 2 ▸ *f*) and structural changes were observed in the N- and C-terminal regions.

### 3.2. Haem structures   

**Figure 3:**
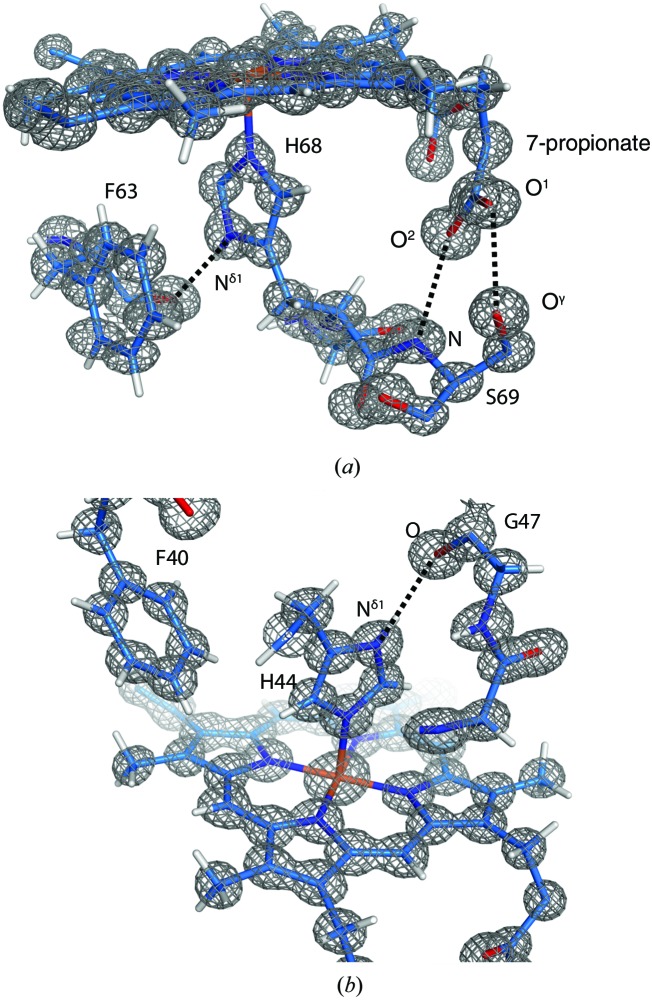
Structure around the axial ligands. (*a*) A 2*F*_o_ − *F*_c_ map around His68 is shown as a grey mesh contoured at the 2.0σ level in the oxidized form 1 structure. Dashed lines indicate hydrogen bonds between His68 N^δ1^ and Phe63 O and between Ser69 and the 7-propionate. (*b*) A 2*F*_o_ − *F*_c_ map around His44 is shown as a grey mesh contoured at the 2.0σ level in the oxidized form 1 structure. The dashed line indicates the hydrogen bond between His44 N^δ1^ and Gly47 O.

**Figure 4:**
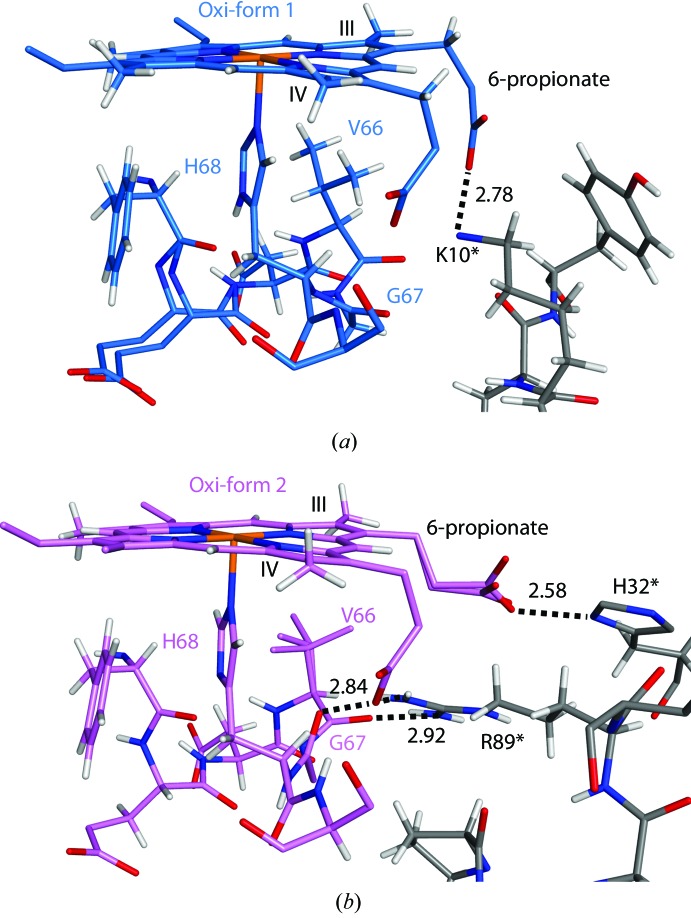
Interactions with neighbouring molecules in the form 1 and form 2 crystals around His68. (*a*) The 6-propionate group interacts with Lys10* of the neighbouring molecule in the crystal in the oxidized form 1 structure. (*b*) The 6-propionate group interacts with His32* in the oxidized form 2 structure. The main-chain carbonyl O atoms of Val66 and Gly67 also interact with Arg89*. The C atoms of the neighbouring molecules in the crystals are coloured grey in (*a*) and (*b*). Bond distances between N and O atoms (in Å) are indicated in the figures.

The 7-propionate group forms hydrogen bonds to the side-chain hydroxyl and main-chain amide of Ser69 in both crystal forms (Fig. 3 ▸ *a*). Strong peaks in the haem OMIT maps clearly indicated the atomic positions of the carboxyl groups of the 7-propionate (Fig. 1 ▸). Restraints on bond angles and lengths were removed for the carboxyl groups of the 7-propionate in *SHELXL* refinement. The C—O distances of the carboxyl groups (C—O^1^ and C—O^2^) are listed in Table 2 ▸. In the form 1 structures, the 6-propionate group makes a contact with the N^ζ^ atom of Lys10* (Fig. 4 ▸ *a*; residues of the neighbouring molecule in the crystal are hereafter indicated with an asterisk). In the form 2 structures, the 6-propionate group interacts with the N^δ1^ atom of His32* (Fig. 4 ▸ *b*). Superposition on the porphyrin-ring atoms shows that the haem-plane conformations are quite similar among the four structures (Supplementary Fig. S3). The 7-propionate groups also show a quite similar conformation, while large differences are observed in the conformations of the 6-propionate groups.

### 3.3. Structures of haem axial ligands   

The distances and angles involved in the haem iron coordination do not show significant changes between the oxidized and reduced forms (Table 3 ▸). Only a small difference of 0.03 Å is observed in the distance from Fe to His68 N^∊2^ between the oxidized form 1 and reduced form 1 structures. The differences in the other iron-coordination distances are less than 0.03 Å between the oxidized and reduced structures. In a comparison of the form 1 and form 2 structures, most of the iron-coordination distances and angles also show small differences, but relatively large changes of an ∼8° rotation are observed in the angles between the imidazole plane of His68 and the N_II_—N_IV_ axis of the haem plane.

The N^δ1^ atoms of the two axial ligands His44 and His68 form hydrogen bonds to the main-chain carbonyl O atoms of Gly47 and Phe63, respectively (Fig. 3 ▸). Supplementary Figs. S4 and S5 show the hydrogen OMIT maps of the axial ligands His44 and His68, respectively. The hydrogen-bond distances from His44 N^δ1^ to Gly47 O are 2.852 (7) Å in the oxidized form 1 structure and 2.868 (6) Å in the reduced form 1 structure. The hydrogen-bond distance from His68 N^δ1^ to Phe63 O is 2.893 (6) Å in the oxidized form 1 structure and 3.097 (5) Å in the reduced form 1 structure. If the final models did not contain the H atoms of His44 and His68, the H atoms in the imidazole rings of His44 and His68 except for the H^∊2^ atoms were generated by the riding model of *SHELXL* to estimate the standard deviations for hydrogen-bond distances from His44 to Gly47 and from His68 to Phe63.

### 3.4. Differences between two crystal forms   

**Figure 5:**
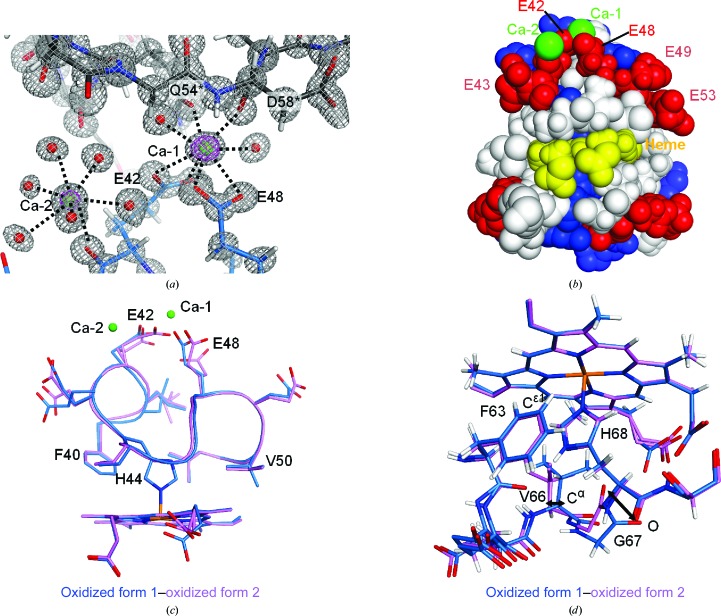
The calcium-binding sites and structural comparison around the axial ligands between the form 1 and form 2 structures. (*a*) The calcium-binding sites in the oxidized form 1 structure. Ca^2+^ ions are shown as green spheres and water molecules involved in calcium coordination are shown as red spheres. The C atoms of the neighbouring molecule in the crystal are coloured grey. 2*F*_o_ − *F*_c_ maps are shown as grey and pink meshes contoured at the 2.0σ and 10.0σ levels, respectively. (*b*) The protein surface around the haem propionate groups is shown as a space-filling model. Acidic residues (Asp and Glu) and basic residues (Arg, His and Lys) are shown in red and blue, respectively. Other residues are shown in white and the haem group is shown in yellow. Ca^2+^ ions are shown in green. (*c*) Structure comparison around the axial ligand His44 between the form 1 (blue) and form 2 (pink) structures of the oxidized form. Main-chain atoms are shown as tubes and side-chain atoms are depicted in stick representation. Ca^2+^ ions are shown as green spheres. (*d*) Structure comparison around the axial ligand His68 between the form 1 (blue) and form 2 (pink) structures of the oxidized form. The shifts of Val66 and Gly67 are shown as double-headed arrows. (*c*) and (*d*) show the superposition of the atoms in the porphyrin rings.

Two Ca^2+^ ions are found in the form 1 structures (Fig. 5 ▸ *a*). The calcium-binding sites are located on the protein surface, where acidic residues are clustered around the exposed haem propionate groups (Fig. 5 ▸ *b*). One of the Ca^2+^ ions (Ca-1) is coordinated by the side-chain carboxyl groups of two glutamic acid residues, Glu42 and Glu48, located around the axial ligand His44. Ca-1 is also coordinated by the side-chain amide O atom of Gln54* and the main-chain carbonyl O atom of Asp58*, both of which are residues of the neighbouring molecule in the crystal. In addition, two water molecules are involved in coordination of the Ca-1 ion. Another Ca ion (Ca-2) is coordinated by the main-chain carbonyl O atom of Glu42 and water molecules; Ca-2 makes no direct interaction with the neighbouring molecules in the crystal. The interaction mediated by Ca-1 changes the side-chain structure around His44, but His44 does not show significant changes in the haem iron coordination between the form 1 and form 2 structures (Fig. 5 ▸ *c*). Structural comparison between the form 1 and form 2 structures shows a 2.2° rotation of the aromatic ring of Phe40 and a 0.45 Å shift of the C^α^ atom of Val50.

In the form 2 structures, Arg89* of the neighbouring molecule in the crystal interacts with both Val66 and Gly67 next to the axial ligand His68 (Fig. 4 ▸ *b*). The interaction changes the conformation of Gly67, and the movement of the main-chain carbonyl O atoms is 2.80 Å between the form 1 and form 2 structures (Fig. 5 ▸ *d*). The position of Val66 is also changed by the interaction with Arg89*. The movement of the C^α^ atoms of Val66 is 1.38 Å between the form 1 and form 2 structures (Fig. 5 ▸ *d*). The positions of the aromatic ring of Phe63 are also changed by the interaction with the neighbouring molecule in the crystal. The C^∊1^ atoms, which are proximal to the haem Fe atom in the aromatic ring of Phe63, are located at distances of 4.99 and 5.25 Å from the haem Fe atoms in the form 1 and form 2 structures, respectively. Although the packing interaction induces structural changes around the axial ligand His68, there are small changes in the iron-coordination distance of His68 (Table 3 ▸).

## 4. Discussion   

The overall structure of porcine b5 is very similar to the b5 structures from other species, especially regarding the haem-binding region. Superposition of the crystal structures of porcine b5 (oxidized form 2) and bovine b5 (PDB entry 1cyo; Durley & Mathews, 1996 ▸) shows a root-mean-square distance of 0.45 Å for 84 C^α^ atoms (Supplementary Fig. S6). In the solution structures of mammalian microsomal b5, the N- and C-terminal regions appear as highly flexible structures (Banci *et al.*, 1997 ▸, 2000 ▸; Arnesano *et al.*, 1998 ▸; Zhang *et al.*, 2004 ▸). In the crystal structures of porcine b5, flexible structures appear as several multiple conformations observed in the N- and C-terminal residues, and the seven disordered residues at the N-terminus also indicate flexibility. The high-resolution structures of porcine b5 reveal multiple conformations that were not observed in the lower resolution structure of bovine b5.

Form 1 and 2 crystals were obtained at pH 7.4 and 5.5, respectively. In a pulse voltammetry experiment using bovine b5, the values of the redox potential were decreased at increasing pH values (Qian *et al.*, 2002 ▸). Structural changes between the different crystal forms are observed near the axial ligand His68 (Fig. 5 ▸ *d*) and in the conformation of the 6-propionate group (Fig. 4 ▸). The structure around His68 is affected by interactions with the neighbouring molecules in the form 2 crystal (Fig. 4 ▸ *b*). For the 6-propionate group, restrained refinement was performed for the oxidized form 2, reduced form 1 and reduced form 2 structures. The 6-propionate groups showed multiple conformations in the oxidized form 2 and reduced form 1 structures (Supplementary Fig. S3), and the electron density of the 6-propionate group was ambiguous in the reduced form 2 structure. From these results, it is difficult to estimate pH-dependent changes from the axial ligands and the 6-propionate group. On the other hand, the 7-propionate groups show a highly similar conformation in the crystal structures of porcine and bovine b5s (Supplementary Figs. S3 and S6*b*). Hydrogen bonds to Ser69 fix the conformation of the 7-propionate, although the 7-propionate group could form an extended conformation in the four crystals because there was sufficient space between adjacent molecules. The sub-angstrom data for porcine b5 allowed unrestrained refinement of the bond angles and lengths of the atoms in the 7-propionate group (Table 2 ▸). The C—O bond distances of the carboxyl groups of the 7-propionate indicate that C—O^1^ is a double bond in the reduced form 1 structure (the typical C=O double-bond distance is 1.21 Å). However, the C—O^2^ distance is ∼0.07 Å shorter than the typical distance for a protonated C—OH bond (1.32 Å). Therefore, the protonation state in the reduced form 1 structure is unclear. On the other hand, the uniform C—O distances in the oxidized form 1 structure indicate that the 7-propionate group is in a charged state. Unrestrained refinement was also applied to the 6-propionate group in the oxidized form 1 structure. The C—O^1^ [1.253 (9) Å] and C—O^2^ [1.246 (10) Å] distances are consistent with the results for the 7-propionate group. In the reduced form 2 structure, the C—O bond distances of the 7-propionate indicate that the O^1^ atom is possibly protonated. Form 2 crystals were obtained at pH 5.5, which is lower than the experimentally measured p*K*_a_ values of 5.7 and 5.9 for oxidized and reduced haem, respectively (Das & Medhi, 1998 ▸). On the other hand, the carboxyl group in the oxidized form 2 structure is in a charged state as deduced from the C—O bond distances of the 7-propionate. Based on the results of electrostatics calculations, Mao *et al.* (2003 ▸) reported that haem oxidation is coupled to some proton loss. The protonation states in the form 2 structures (determined at pH 5.5) are consistent with this result.

**Figure 6:**
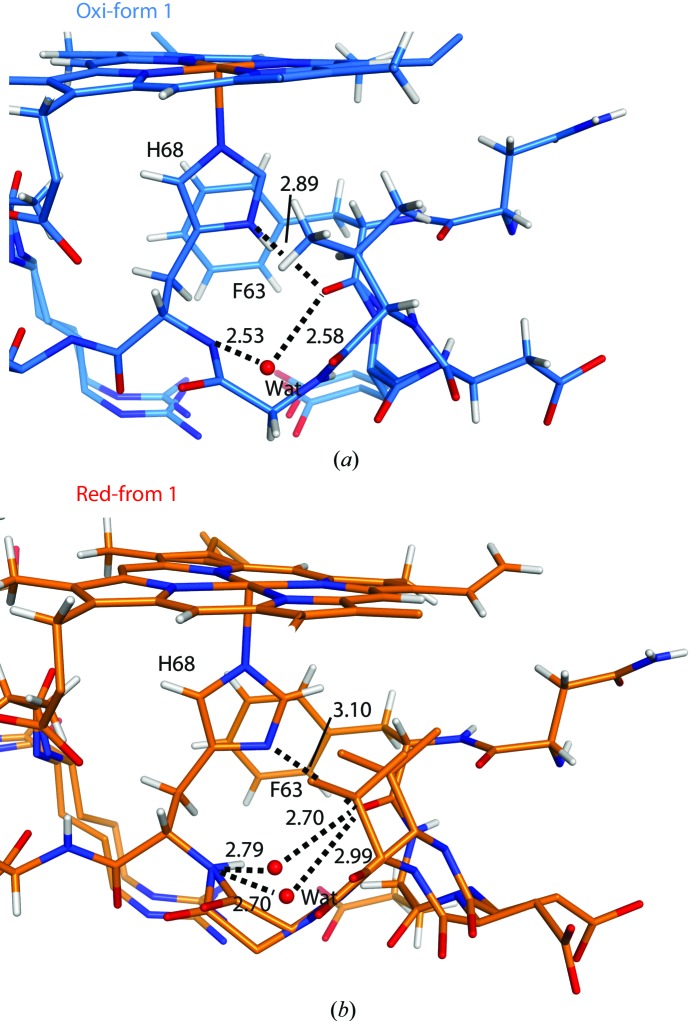
Hydrogen bonds around the axial ligands. The structures around the axial ligand His68 (*a*) in the oxidized form 1 structure and (*b*) in the reduced form 1 structure. The amino-acid residues and the haem molecule are shown as stick models and water molecules are shown as spheres. The water molecule shows an alternate conformation in the reduced form 1 structure. Dashed lines indicate hydrogen bonds between the amino-acid residues and water molecules and between His68 and Phe63. The bond distances between the N^δ1^ atom of His68 and the carbonyl O atom of Phe63, between the amide N atom of His68 and the O atom of water, and between the carbonyl O atom of Phe63 and the O atom of water (in Å) are indicated in the figures.

Structural changes depending on the redox state have been investigated in haem-binding proteins. X-ray absorption spectroscopy showed that the bond distance from the haem Fe to the proximal His changed by 0.15 Å between the oxidized and reduced forms of myoglobin (Longa *et al.*, 2003 ▸). On the other hand, apparent structural changes were not observed between the oxidized and reduced forms in solution structures of cytochrome *c* (Feng *et al.*, 1990 ▸). In the high-resolution structures of porcine b5, significant changes could not be detected in the haem iron-coordination geometry when the oxidized and reduced structures were compared in the same crystal form (Table 3 ▸). The stabilization of b5 was decreased by mutation of the axial ligand (Wang *et al.*, 2003 ▸). This result suggests that structural changes of the axial ligands are highly restricted in b5. The hydrogen-bond distance from His44 to Gly47 also exhibited a small difference of ∼0.02 Å between the form 1 structures. However, there was an ∼0.20 Å difference in the hydrogen-bond distance from His68 N^δ1^ to the main-chain carbonyl O atom of Phe63 between the form 1 structures. The residues from Phe63 to His68 make a few interactions with the neighbouring molecules in the form 1 crystal. Because there is a relatively large space between adjacent molecules, the residues show flexible structures, as observed from the multiple conformations of Glu64–Gly67 (Fig. 6 ▸). In the form 2 structures, Arg89* of the neighbouring molecule in the crystal interacts with both Val66 and Gly67 (Fig. 4 ▸ *b*). The interactions with Arg89* and other interactions with the neighbouring molecules in the crystal fix the structure from Phe63 to His68, and small spaces exist between the adjacent molecules. In addition, water molecules were found near the main-chain atoms of Phe63 and His68 in the form 1 structures (Fig. 6 ▸). In the reduced form 1 structure, the water molecules show an alternate conformation (Fig. 6 ▸ *b*). The water molecules form hydrogen bonds to both the main-chain carbonyl of Phe63 and the main-chain amide of His68. The hydrogen-bond distances in the oxidized form 1 structure are shorter (0.2–0.4 Å) than those in the reduced form 1 structure. These results indicate that the hydrogen-bond network around His68 might be involved in regulating the redox state of the haem if the residues from Phe63 to His68 have flexible structures. The changes in the hydrogen-bond network around the axial ligand are consistent with the structural and mutation studies of haem proteins, which suggested hydrogen bonding to the axial ligand as an important factor in controlling the haem redox potential (Poulos, 1996 ▸).

Divalent cations (Ca^2+^ or Mg^2+^) have been reported to reduce the rate of b5 reduction by b5R (Tamura *et al.*, 1988 ▸). In the form 1 structure the Ca^2+^ ions are coordinated by the glutamic acid residues Glu42 and Glu48 located on the surface near the haem propionate group (Fig. 5 ▸ *b*). These glutamic acid residues are conserved in mammalian b5 (Supplementary Fig. S2). Studies using the b5–b5R docking model have indicated that the acidic surface residue Glu48 of b5 interacts with the basic surface residue Lys97 near the FAD- and NADH-binding sites of b5R (Nishida & Miki, 1996 ▸). Therefore, the binding of Ca ions may prevent the electrostatic interaction between acidic surface residues of b5 and basic surface residues of b5R.

## Supplementary Material

PDB reference: solubilized domain of porcine cytochrome *b*_5_, oxidized form, crystal form 1, 3x32

PDB reference: crystal form 2, 3x33

PDB reference: reduced form, crystal form 1, 3x34

PDB reference: crystal form 2, 3x35

Supporting Information.. DOI: 10.1107/S1399004715009438/mh5178sup1.pdf

## Figures and Tables

**Table 1:** Diffraction data and refinement statistics Values in parentheses are for the highest resolution shell.

	Oxidized form 1	Oxidized form 2	Reduced form 1	Reduced form 2
Diffraction data				
Diffraction source	BL17A, PF	BL17A, PF	BL5A, PF	BL5A, PF
Wavelength ()	0.91	0.91	0.80	0.90
Temperature (K)	100	100	100	100
Detector	ADSC Q270	ADSC Q270	ADSC Q315	ADSC Q315
Space group	*P*2_1_2_1_2_1_	*P*2_1_2_1_2_1_	*P*2_1_2_1_2_1_	*P*2_1_2_1_2_1_
*a*, *b*, *c* ()	32.3, 39.3, 61.0	33.6, 51.2, 55.1	33.6, 38.9, 60.6	33.7, 51.3, 55.3
, , ()	90, 90, 90	90, 90, 90	90, 90, 90	90, 90, 90
Resolution range ()	500.83 (0.840.83)	500.93 (0.950.93)	500.76 (0.770.76)	500.95 (0.970.95)
Total No. of reflections	1130607	410023	1237307	423970
No. of unique reflections	72789	63824	99196	58020
Completeness (%)	98.2 (82.3)	98.8 (96.7)	99.8 (97.3)	95.8 (87.0)
Multiplicity	15.5 (10.8)	6.4 (3.5)	12.5 (6.5)	7.3 (3.3)
*I*/(*I*)	59.8 (7.6)	50.9 (3.5)	67.4 (4.1)	54.8 (2.3)
*R*_r.i.m._ † (%)	8.9 (37.4)	6.8 (43.5)	7.1 (38.6)	8.4 (43.9)
Overall *B* factor from Wilson plot (^2^)	6.2	8.4	6.5	8.8
Refinement				
Resolution range ()	500.83	500.93	500.76	500.95
Final *R*_cryst_ (%)	10.8	12.9	10.6	13.9
Final *R*_free_ (%)	12.7	16.9	13.2	18.1
No. of non-H atoms				
Protein	867	898	862	893
Ca^2+^	2		2	
HEPES	15			
Acetate		4		4
Haem	43	47	51	43
Water	214	211	252	200
No. of H atoms				
Protein	416	232	419	150
Haem	20	13	17	2
Average *B* factors (^2^)				
Protein	10.0	15.3	9.7	16.1
Haem	9.6	12.5	9.1	12.8
Ca^2+^	6.4		5.9	
HEPES	12.4			
Acetate		15.6		14.9
Water	18.1	24.6	18.3	28.6
Ramachandran plot				
Most favoured (%)	90.9	92.2	92.2	93.5
Allowed (%)	9.1	7.8	7.8	6.5

† *R*_r.i.m._ = 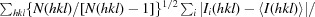 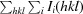, where *N*(*hkl*) is the data multiplicity.

**Table 2:** CO bond distances in the 7-propionate group of haem (in ) The values in parentheses represent the estimated standard deviations derived from least-squares refinement using *SHELXL*.

	Oxidized form 1	Oxidized form 2	Reduced form 1	Reduced form 2
CO^1^	1.252 (9)	1.273 (17)	1.225 (10)	1.289 (17)
CO^2^	1.251 (8)	1.278 (16)	1.252 (9)	1.237 (19)

**Table 3:** Haem iron-coordination geometry The values in parentheses represent the estimated standard deviations derived from least-squares refinement using *SHELXL*.

	Oxidized form 1	Oxidized form 2	Reduced form 1	Reduced form 2	|Oxidized reduced| form 1	|Oxidized reduced| form 2	Oxidized |form 1 form 2|	Reduced |form 1 form 2|
Distances ()								
FeHis44N^2^	2.004 (4)	1.996 (9)	2.013 (3)	1.984 (11)	0.009	0.012	0.008	0.029
FeHis68N^2^	1.992 (5)	1.975 (8)	2.022 (4)	1.970 (9)	0.030	0.005	0.017	0.052
FeN_I_	1.996 (6)	2.008 (8)	2.012 (4)	2.020 (9)	0.016	0.012	0.012	0.008
FeN_II_	1.986 (5)	1.997 (7)	2.003 (3)	1.997 (8)	0.017	0.000	0.011	0.006
FeN_III_	1.979 (6)	1.961 (8)	1.999 (4)	1.955 (9)	0.020	0.006	0.018	0.044
FeN_IV_	2.004 (5)	1.998 (7)	1.997 (3)	1.995 (8)	0.007	0.003	0.006	0.002
Angles ()								
His44 N^2^FeHis68 N^2^	177.9 (2)	177.9 (3)	178.6 (1)	177.7 (4)	0.7	0.2	0.0	0.9
N_I_FeN_III_	179.2 (2)	179.2 (3)	179.3 (2)	179.1 (4)	0.1	0.1	0.0	0.2
N_II_FeN_IV_	178.9 (2)	179.1 (3)	179.5 (1)	178.3 (4)	0.6	0.8	0.2	1.2
His44 imidazole(N_II_N_IV_)†	46.5	45.1	46.9	46.0	0.4	0.9	1.4	0.9
His68 imidazole(N_II_N_IV_)†	17.9	25.8	18.1	26.1	0.2	0.3	7.9	8.0

† The angle of the His imidazole plane against the N_II_N_IV_ axis on the haem plane. Positive and negative values represent the clockwise and counterclockwise directions to the N_II_N_IV_ axis viewing from the axial ligands.

## References

[bb1] Adams, P. D. *et al.* (2010). *Acta Cryst.* D**66**, 213–221.

[bb2] Altuve, A., Wang, L., Benson, D. R. & Rivera, M. (2004). *Biochem. Biophys. Res. Commun.* **314**, 602–609. 10.1016/j.bbrc.2003.12.138 14733950

[bb3] Arnesano, F., Banci, L., Bertini, I. & Felli, I. C. (1998). *Biochemistry*, **37**, 173–184. 10.1021/bi971896w 9425037

[bb4] Banci, L., Bertini, I., Ferroni, F. & Rosato, A. (1997). *Eur. J. Biochem.* **249**, 270–279. 10.1111/j.1432-1033.1997.t01-1-00270.x 9363779

[bb5] Banci, L., Bertini, I., Rosato, A. & Scacchieri, S. (2000). *Eur. J. Biochem.* **267**, 755–766. 10.1046/j.1432-1327.2000.01054.x 10651812

[bb6] Das, D. K. & Medhi, O. K. (1998). *J. Inorg. Biochem.* **70**, 83–90. 10.1016/s0162-0134(98)10002-8 9666570

[bb7] DeLano, W. L. (2002). *PyMOL*. http://www.pymol.org.

[bb8] Della Longa, S., Arcovito, A., Benfatto, M., Congiu-Castellano, A., Girasole, M., Hazemann, J. L. & Lo Bosco, A. (2003). *Biophys. J.* **85**, 549–558. 10.1016/S0006-3495(03)74499-3 PMC1303110 12829509

[bb9] Durley, R. C. E. & Mathews, F. S. (1996). *Acta Cryst.* D**52**, 65–76. 10.1107/S0907444995007827 15299727

[bb10] Emsley, P., Lohkamp, B., Scott, W. G. & Cowtan, K. (2010). *Acta Cryst.* D**66**, 486–501. 10.1107/S0907444910007493 PMC2852313 20383002

[bb11] Feng, Y., Roder, H. & Englander, S. W. (1990). *Biochemistry*, **29**, 3494–3504. 10.1021/bi00466a011 2162193

[bb12] Fukushima, H., Grinstead, G. F. & Gaylor, L. (1981). *J. Biol. Chem.* **256**, 4822–4826. 7228857

[bb13] Funk, W. D., Lo, T. P., Mauk, M. R., Brayer, G. D., MacGillivray, R. T. A. & Mauk, A. G. (1990). *Biochemistry*, **29**, 5500–5508. 10.1021/bi00475a013 2117468

[bb14] Gan, J.-H., Wu, J., Wang, Z.-Q., Wang, Y.-H., Huang, Z.-X. & Xia, Z.-X. (2002). *Acta Cryst.* D**58**, 1298–1306. 10.1107/s0907444902010016 12136141

[bb15] Kimura, S., Kawamura, M. & Iyanagi, T. (2003). *J. Biol. Chem.* **278**, 3580–3589. 10.1074/jbc.M209838200 12459552

[bb16] Mao, J., Hauser, K. & Gunner, M. R. (2003). *Biochemistry*, **42**, 9829–9840. 10.1021/bi027288k 12924932

[bb17] Mitoma, J. & Ito, A. (1992). *EMBO J.* **11**, 4197–4203. 10.1002/j.1460-2075.1992.tb05513.x PMC556930 1396600

[bb18] Nishida, H. & Miki, K. (1996). *Proteins*, **26**, 32–41. 10.1002/(SICI)1097-0134(199609)26:1<32::AID-PROT3>3.0.CO;2-I 8880927

[bb19] Otwinowski, Z. & Minor, W. (1997). *Methods Enzymol.* **276**, 307–326. 10.1016/S0076-6879(97)76066-X 27754618

[bb20] Paithankar, K. S., Owen, R. L. & Garman, E. F. (2009). *J. Synchrotron Rad.* **16**, 152–162. 10.1107/S0909049508040430 19240327

[bb21] Porter, T. D. (2002). *J. Biochem. Mol. Toxicol.* **16**, 311–316. 10.1002/jbt.10052 12481306

[bb22] Poulos, T. L. (1996). *J. Biol. Inorg. Chem.* **1**, 356–359.

[bb24] Qian, C., Yao, Y., Ye, K., Wang, J., Tang, W., Wang, Y., Wang, W., Lu, J., Xie, Y. & Huang, Z. (2001). *Protein Sci.* **10**, 2451–2459. 10.1110/ps.12401 PMC2374031 11714912

[bb23] Qian, W., Wang, Y.-H., Wang, W.-H., Yao, P., Zhuang, J.-H., Xie, Y. & Huang, Z.-X. (2002). *J. Electroanal. Chem.* **535**, 85–96.

[bb25] Ren, Y., Wang, W.-H., Wang, Y.-H., Case, M., Qian, W., McLendon, G. & Huang, Z.-X. (2004). *Biochemistry*, **43**, 3527–3536. 10.1021/bi036078k 15035623

[bb26] Rivera, M., Barillas-Mury, C., Christensen, K. A., Little, J. W., Wells, M. A. & Walker, F. A. (1992). *Biochemistry*, **31**, 12233–12240. 10.1021/bi00163a037 1333795

[bb27] Sheldrick, G. M. (2008). *Acta Cryst.* A**64**, 112–122. 10.1107/S0108767307043930 18156677

[bb28] Spatz, L. & Strittmatter, P. (1973). *J. Biol. Chem.* **248**, 793–799. 4346350

[bb29] Sun, Y.-L., Wang, Y.-H., Yan, M.-M., Sun, B.-Y., Xie, Y., Huang, Z.-X., Jiang, S.-K. & Wu, H.-M. (1999). *J. Mol. Biol.* **285**, 347–359. 10.1006/jmbi.1998.2295 9878411

[bb30] Tamura, M., Yubisui, T. & Takeshita, M. (1988). *Biochem. J.* **251**, 711–715. 10.1042/bj2510711 PMC1149062 3137923

[bb31] Walker, F. A., Emrick, D., Rivera, J. E., Hanquet, B. J. & Buttlaire, D. H. (1988). *J. Am. Chem. Soc.* **110**, 6234–6240. 10.1021/ja00226a045 22148805

[bb32] Wang, W.-H., Lu, J.-X., Yao, P., Xie, Y. & Huang, Z.-X. (2003). *Protein Eng. Des. Sel.* **16**, 1047–1054. 10.1093/protein/gzg134 14983086

[bb33] Wang, L., Sun, N., Terzyan, S., Zhang, X. & Benson, D. R. (2006). *Biochemistry*, **45**, 13750–13759. 10.1021/bi0615689 17105194

[bb34] Wu, J., Gan, J.-H., Xia, Z.-X., Wang, Y.-H., Wang, W.-H., Xue, L.-L., Xie, Y. & Huang, Z.-X. (2000). *Proteins*, **40**, 249–257. 10.1002/(sici)1097-0134(20000801)40:2<249::aid-prot70>3.0.co;2-h 10842340

[bb35] Wu, Y., Wang, Y., Qian, C., Lu, J., Li, E., Wang, W., Lu, J., Xie, Y., Wang, J., Zhu, D., Huang, Z. & Tang, W. (2001). *Eur. J. Biochem.* **268**, 1620–1630. 11248680

[bb36] Yamada, M., Tamada, T., Takeda, K., Matsumoto, F., Ohno, H., Kosugi, M., Takaba, K., Shoyama, Y., Kimura, S., Kuroki, R. & Miki, K. (2013). *J. Mol. Biol.* **425**, 4295–4306. 10.1016/j.jmb.2013.06.010 23831226

[bb37] Yao, P., Wu, J., Wang, Y.-H., Sun, Y.-H., Xia, Z.-X. & Huang, Z.-X. (2002). *Eur. J. Biochem.* **269**, 4287–4296. 10.1046/j.1432-1033.2002.03120.x 12199707

[bb38] Zhang, Q., Cao, C., Wang, A.-Q., Wang, Y.-H., Wu, H. & Huang, Z.-X. (2004). *Protein Sci.* **13**, 2161–2169. 10.1110/ps.04721104 PMC2279834 15273310

