# Supplementary material for: High-resolution crystal structures of the solubilized domain of porcine cytochrome b 5
Source: Acta Crystallogr D Biol Crystallogr. 2015 Jun 30;71(Pt 7):1572–81. doi: 10.1107/S1399004715009438 (PMC4498607; doi:10.1107/S1399004715009438)
Supplement: Supplementary file 1 [file d-71-01572-sup1.pdf]

BIOLOGICAL CRYSTALLOGRAPHY

Volume 71 (2015)

Supporting information for article:

High-resolution crystal structures of the solubilized domain of porcine cytochrome b_5

Yu Hirano, Shigenobu Kimura and Taro Tamada

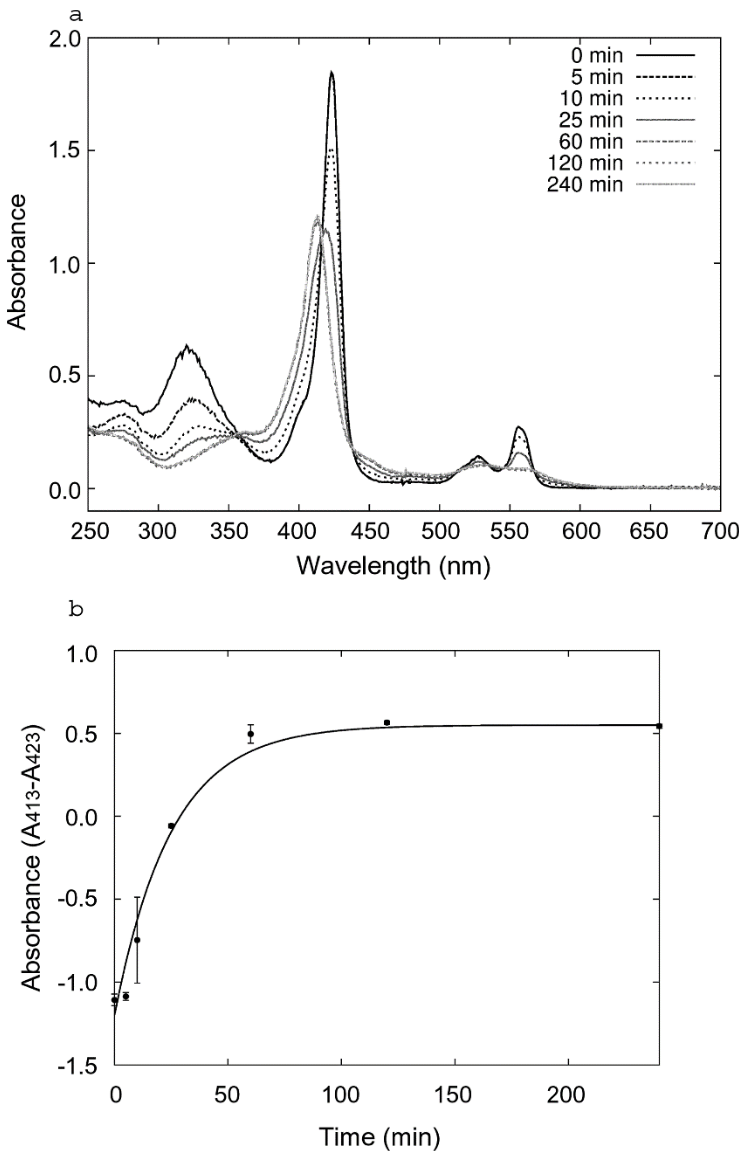

Figure S1 Autoxidation of the reduced form of b5. (a) UV-Vis absorption spectra measured at 0, 5, 10, 25, 60, 120, and 240 min after adding sodium dithionite to the oxidized form of b5 under aerobic conditions. (b) Differences between absorbance at 413 nm (Soret band for the oxidized form) and that at 423 nm (Soret band for the reduced form). The mean values of the triplicate measurements were plotted, and the vertical bars represent the standard deviations. An exponential function fitted to the data was also depicted.

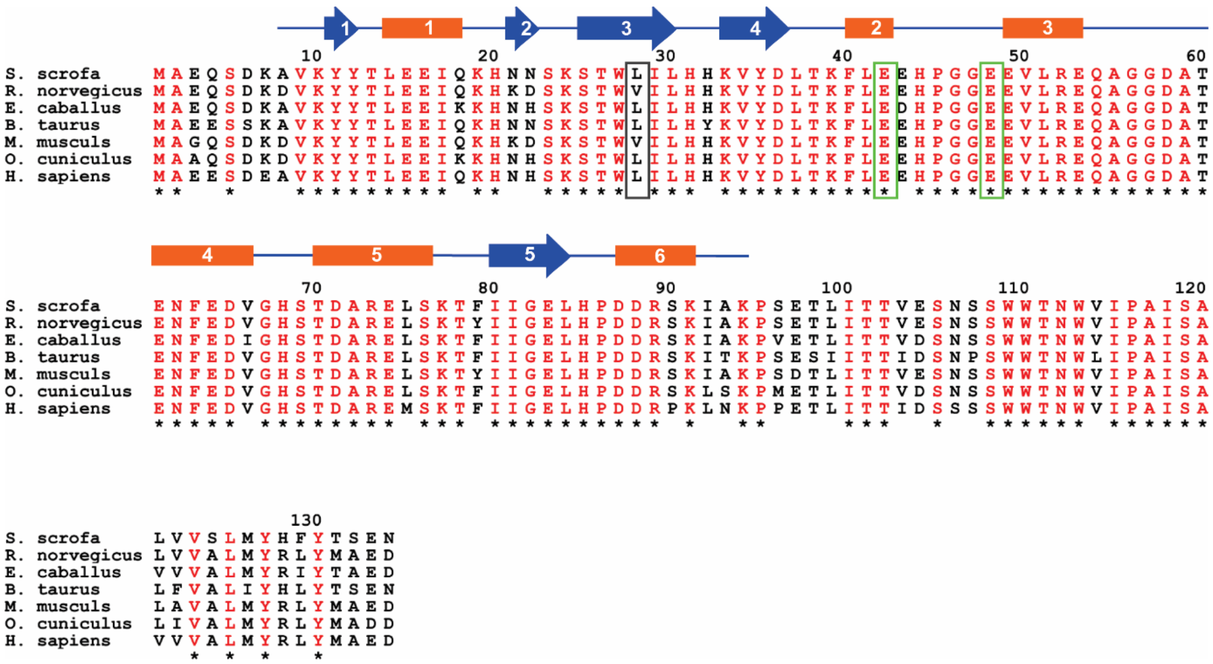

Figure S2 Sequence alignment of mammalian microsomal b5s. Fully conserved residues are shown as red characters. The secondary structure of the oxidized form 2 structure of porcine b5 is shown above the sequence alignment. Glu42 and Glu48 involved in the Ca-coordination are indicated with green boxes. Leu28 at the bottom of the heme binding crevasse is indicated with a gray box. *Sus scrofa*; porcine, *Rattus norvegicus*; rat, *Equus caballus*; horse, *Bos taurus*; bovine, *Mus musculus*; mouse, *Oryctolagus cuniculus*; rabbit, *Homo sapiens*; human.

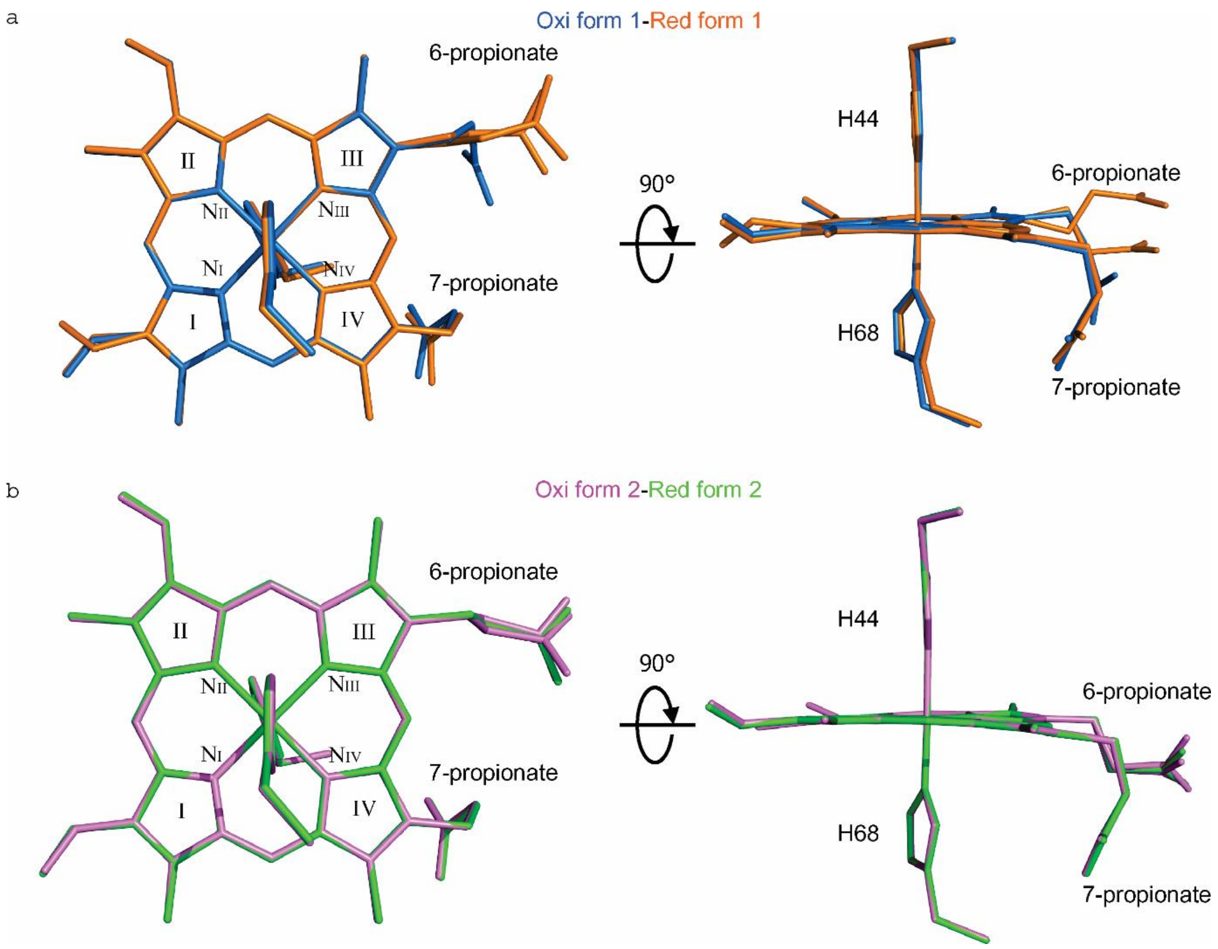

Figure S3 Structure comparison of the heme group. (a) Superposition of the atoms in the porphyrin rings between the oxidized form 1 (blue) and reduced form 1 (orange) structures. (b) Superposition of the atoms in the porphyrin rings between the oxidized form 2 (pink) and reduced form 2 (green) structures. The right panels correspond to the 90° rotation of the left panels around the horizontal axes on the page.

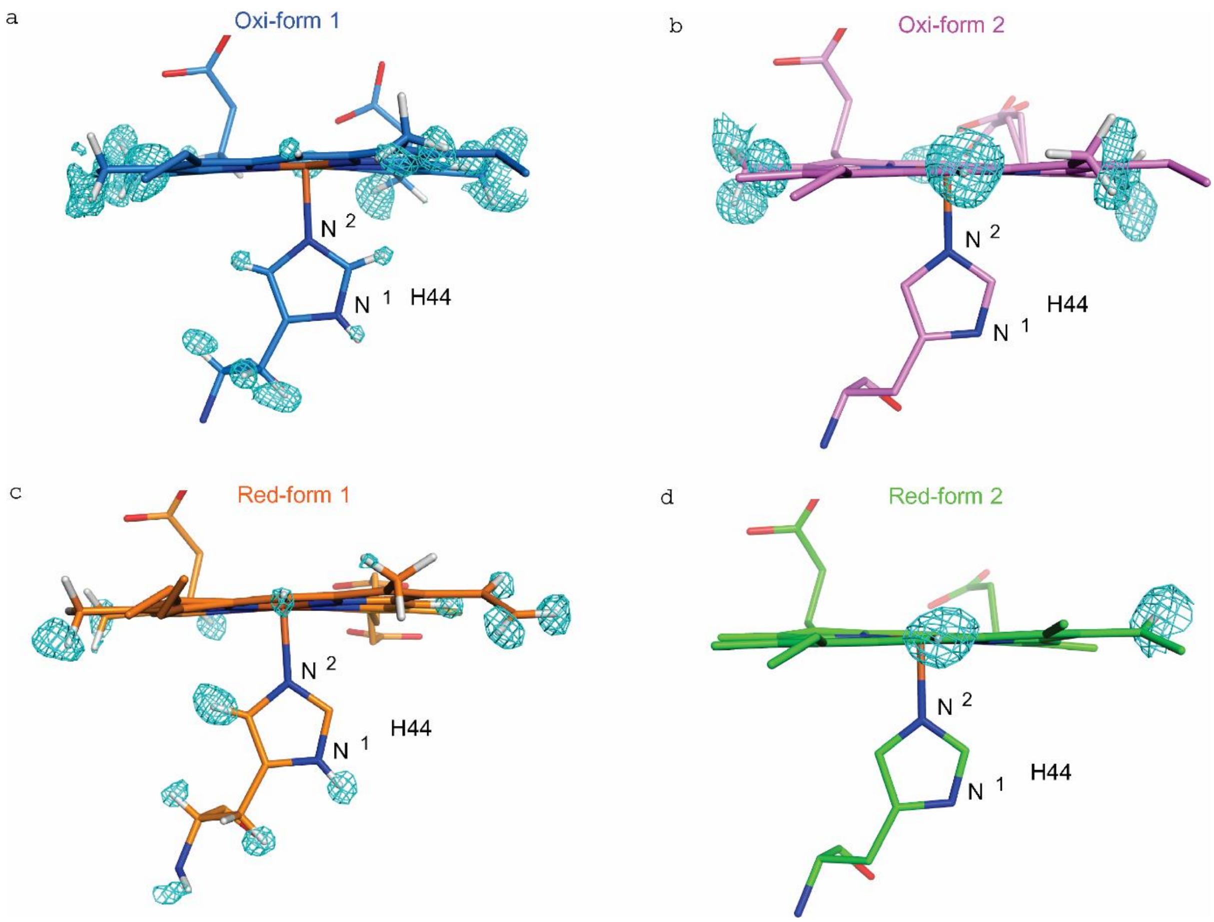

Figure S4 Electron density of hydrogen atoms of His44 and the heme group. F_o-F_c hydrogen omit maps are shown as light blue mesh contoured at the 1.5 σ level (a) in the oxidized form 1 structure, (b) in the oxidized form 2 structure, (c) in the reduced form 1 structure, and (d) in the reduced form 2 structure. The positions of the $N^{\delta1}$ and $N^{\varepsilon2}$ atoms of His44 are indicated in the figures.

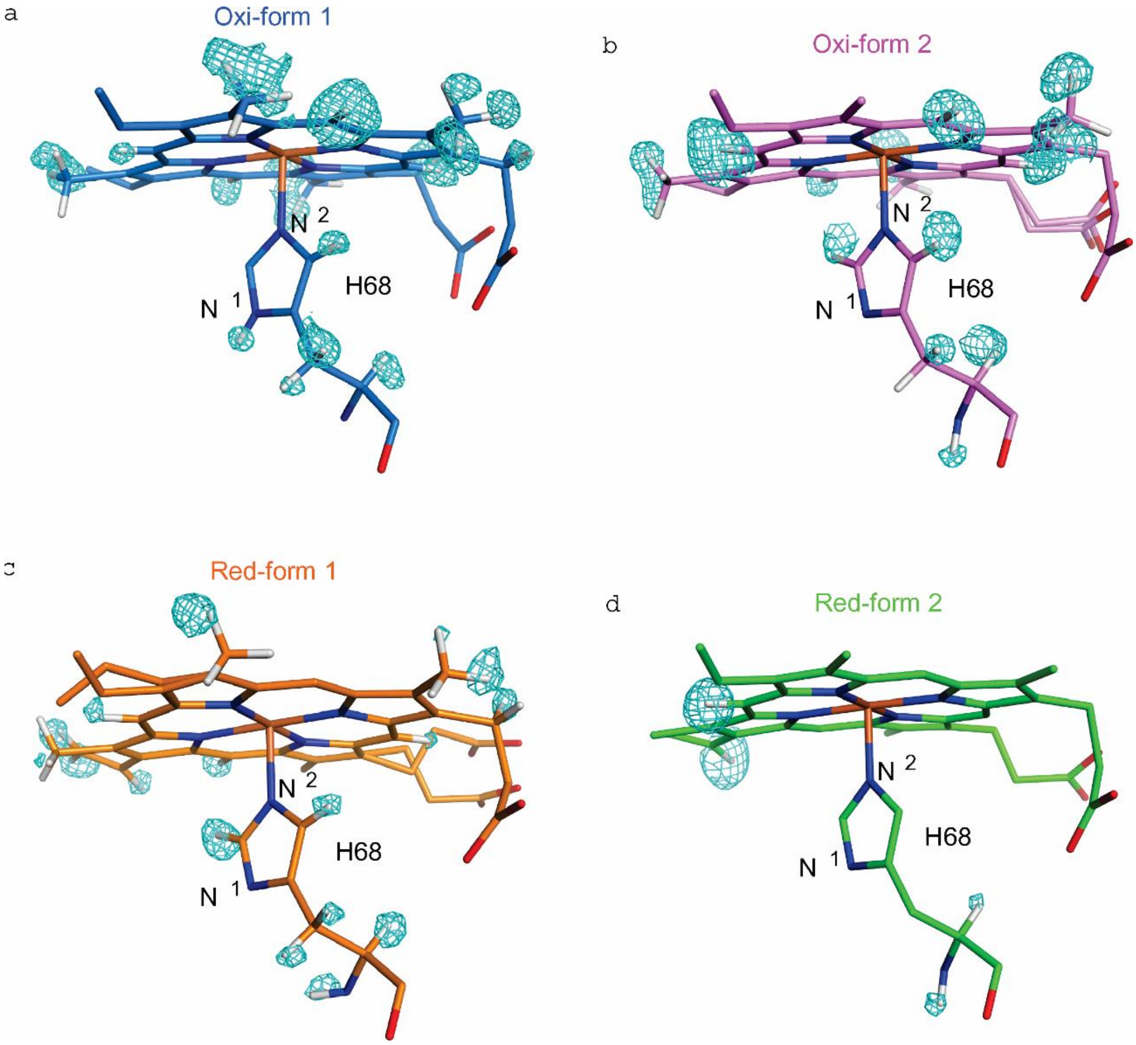

Figure S5 Electron density of hydrogen atoms of His68 and the heme group. F_o-F_c hydrogen omit maps are shown as light blue mesh contoured at the 1.5 σ level (a) in the oxidized form 1 structure, (b) in the oxidized form 2 structure, (c) in the reduced form 1 structure, and (d) in the reduced form 2 structure. The positions of the $N^{\delta 1}$ and $N^{\varepsilon 2}$ atoms of His68 are indicated in the figures.

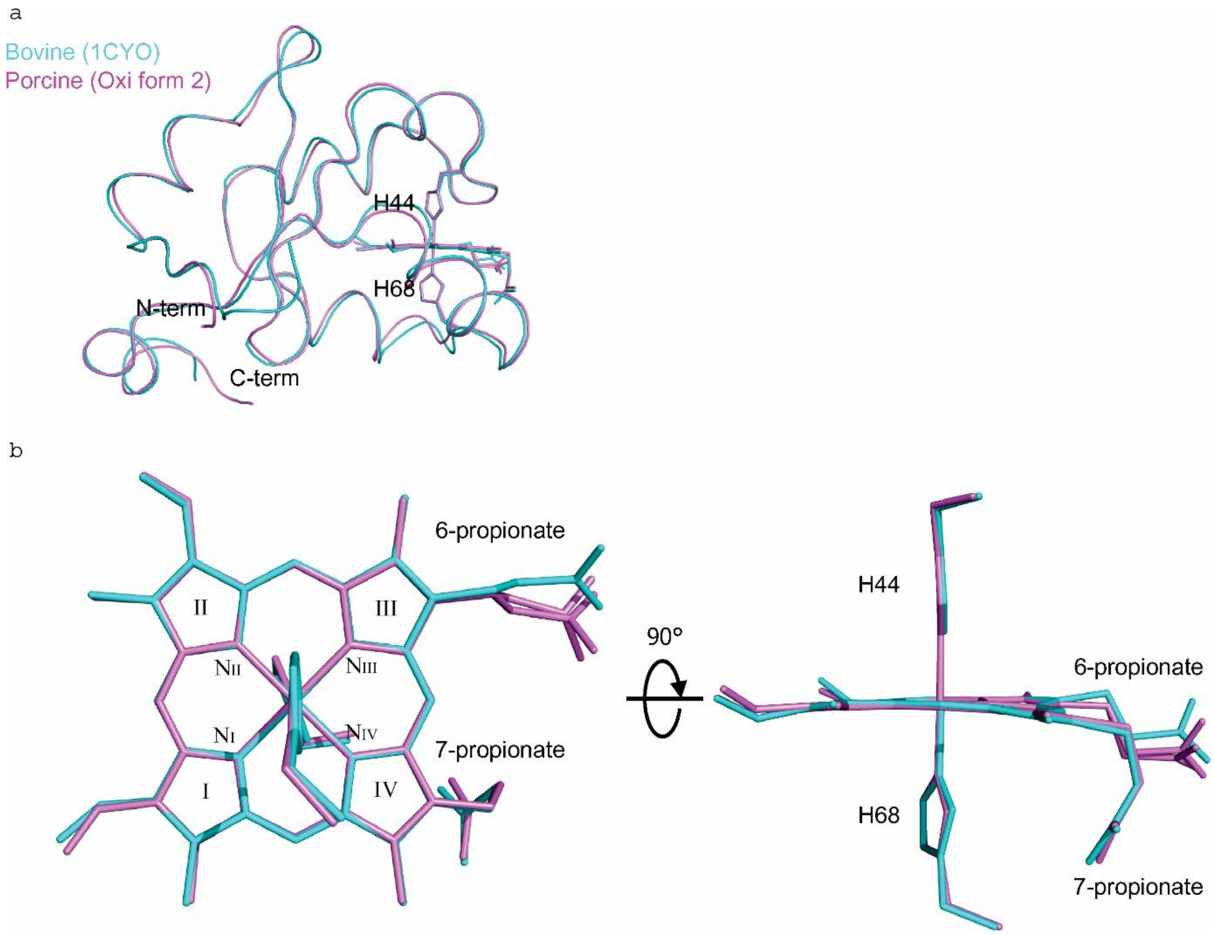

Figure S6 Structure comparison between porcine and bovine b5s. (a) Superposition of C^{α} atoms between the oxidized form 2 structure of porcine b5 (pink) and the crystal structure of bovine b5 (light blue; PDB ID 1CYO). (b) Superposition of the atoms in the porphyrin rings between the oxidized form 2 (pink) and bovine b5 (light blue) structures. The right panel corresponds to the 90° rotation of the left panels around the horizontal axes on the page.

Table S1 Root-mean-square distances among porcine b5 structures (in Å)

	Oxi, form1	Oxi, form2	Red, form1	Red, form2
Oxi, Form 1	-	-	-	-
Oxi, Form 2	0.51	-	-	-
Red, Form 1	0.31	0.66	-	-
Red, Form 2	0.51	0.11	0.67	-
